# Supplementary figures and images for: Gender gap in journal submissions and peer review during the first wave of the COVID-19 pandemic. A study on 2329 Elsevier journals
Source: PLoS One. 2021 Oct 20;16(10):e0257919. doi: 10.1371/journal.pone.0257919 (PMC8528305; doi:10.1371/journal.pone.0257919)

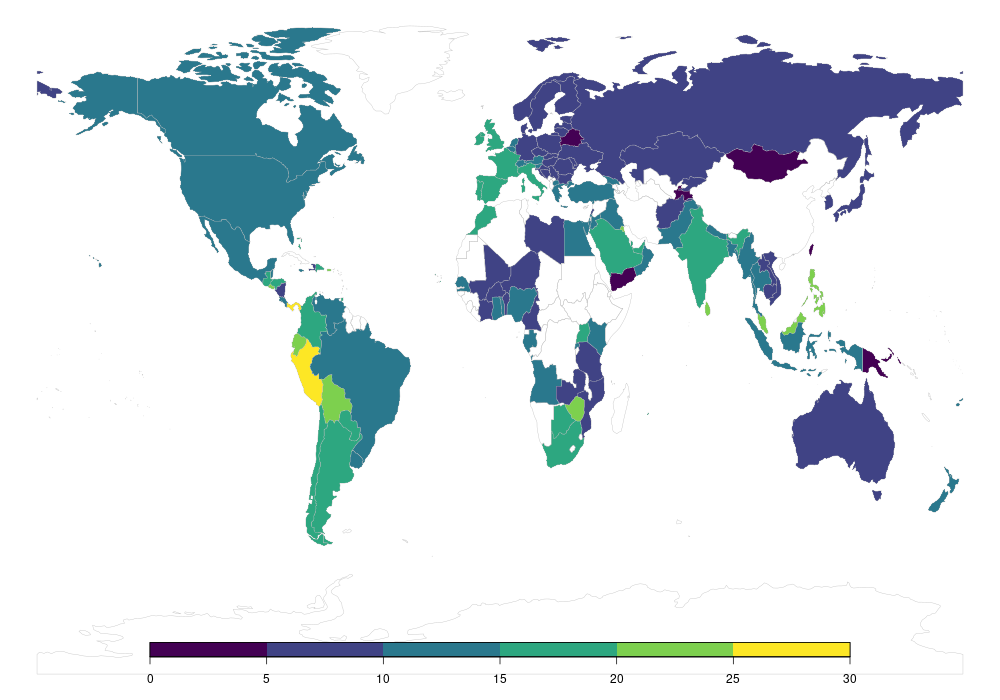

Supplement: S1 Fig — The change was calculated as different rate from the baseline given by median value during the first five weeks of 2020. Data from Google COVID-19 Community Mobility Report (see https://www.google.com/covid19/mobility/; accessed on 30 June 2020). White areas indicate missing data. (TIF) [file pone.0257919.s001.tif]
