# Supplementary material for: Gender gap in journal submissions and peer review during the first wave of the COVID-19 pandemic. A study on 2329 Elsevier journals
Source: PLoS One. 2021 Oct 20;16(10):e0257919. doi: 10.1371/journal.pone.0257919 (PMC8528305; doi:10.1371/journal.pone.0257919)
Supplement: S1 Table — Note that data reported here differ from those in Table 1 because: (i) several authors could have submitted the same manuscript to different journals, which was only counted once here; and (ii) submissions and reviews from academics whose gender was not guessed by our algorithm were included here but not in Table 1. (PDF) [file pone.0257919.s002.pdf]

	Feb-May 2018	Feb-May 2019	Feb-May 2020
HMS submissions	131834	147401	240587
LS submissions	90488	101765	125380
PS submissions	288234	322039	378659
SSE submissions	45109	49480	62823
Total submissions	555665	620685	807449
HMS review invitations	371038	415033	554895
LS review invitations	287028	336203	430908
PS review invitations	770137	986905	1255735
SSE review invitations	77663	109115	139746
Total review invitations	1505866	1847256	2381284
HMS accepted invitations	130723	161447	217064
LS accepted invitations	91643	120589	146120
PS accepted invitations	264307	386956	500644
SSE accepted invitations	33456	51812	67994
Total accepted invitations	520129	720804	931822

Table S1: Total number of new submissions, review invitations, and accepted invitations per area of research in February-May 2020 and corresponding months of 2018 and 2019. Note that data reported here differ from those in Table 1 because: (i) several authors could have submitted the same manuscript to different journals, which was only counted once here; and (ii) submissions and reviews from academics whose gender was not guessed by our algorithm were included here but not in Table 1.
