# Supplementary material for: Gender gap in journal submissions and peer review during the first wave of the COVID-19 pandemic. A study on 2329 Elsevier journals
Source: PLoS One. 2021 Oct 20;16(10):e0257919. doi: 10.1371/journal.pone.0257919 (PMC8528305; doi:10.1371/journal.pone.0257919)
Supplement: S2 Table — The quartiles were calculated using Journal Citation Reportsby Clarivate Analytics. (PDF) [file pone.0257919.s003.pdf]

IF quartile	Health & Medicine	Life Sciences	Physical Sciences & Engineering	Social Sciences & Economics
Q1	15.20	27.30	38.60	35.20
Q2	15.80	26.40	29.60	25.00
Q3	15.10	14.90	11.40	14.80
Q4	5.60	2.60	2.30	1.90
No IF	48.30	28.80	18.10	23.10

Table S2: Proportion (%) of journals included in each quartile of the impact factor distribution by area of research. The quartiles were calculated using The 2020 Journal Citation Reports by Clarivate Analytics
