# Supplementary material for: Gender gap in journal submissions and peer review during the first wave of the COVID-19 pandemic. A study on 2329 Elsevier journals
Source: PLoS One. 2021 Oct 20;16(10):e0257919. doi: 10.1371/journal.pone.0257919 (PMC8528305; doi:10.1371/journal.pone.0257919)
Supplement: S3 Table — Random intercepts included for countries. (PDF) [file pone.0257919.s004.pdf]

	Health & Medicine	Life Sciences	Physical Sciences & Engineering	Social Sciences & Economics
Women	−0.133	−0.054	−0.105	−0.063
	(0.027)	(0.037)	(0.066)	(0.044)
	$p < 0.001$	$p = 0.141$	$p = 0.114$	$p = 0.149$
Age	0.003	−0.001	−0.008	−0.003
	(0.001)	(0.001)	(0.002)	(0.001)
	$p = 0.001$	$p = 0.241$	$p < 0.001$	$p = 0.064$
Women×Age	−0.001	−0.005	−0.005	−0.004
	(0.002)	(0.002)	(0.004)	(0.003)
	$p = 0.392$	$p = 0.029$	$p = 0.216$	$p = 0.212$
Intercept	1.421	1.025	1.338	1.263
	(0.037)	(0.044)	(0.059)	(0.033)
	$p < 0.001$	$p < 0.001$	$p < 0.001$	$p < 0.001$
Observations	51916	11557	5401	3039
Log Likelihood	−99295	−17136	−8891	−3102

Table S3: Mixed effects models predicting February-May 2020 changes in the number of submissions of Covid-related manuscripts per area of research. Random intercepts included for countries.
