# Supplementary material for: Gender gap in journal submissions and peer review during the first wave of the COVID-19 pandemic. A study on 2329 Elsevier journals
Source: PLoS One. 2021 Oct 20;16(10):e0257919. doi: 10.1371/journal.pone.0257919 (PMC8528305; doi:10.1371/journal.pone.0257919)
Supplement: S4 Table — The baseline is represented by the average of corresponding months in 2018 and 2019. Random intercepts included for countries. (PDF) [file pone.0257919.s005.pdf]

	Health & Medicine	Life Sciences	Physical Sciences & Engineering	Social Sciences & Economics
Women	−0.109	−0.066	−0.086	−0.068
	(0.006)	(0.007)	(0.008)	(0.011)
	$p < 0.001$	$p < 0.001$	$p < 0.001$	$p < 0.001$
Age	−0.002	−0.001	−0.003	−0.004
	(0.0002)	(0.0002)	(0.0002)	(0.0004)
	$p < 0.001$	$p < 0.001$	$p < 0.001$	$p < 0.001$
Women×Age	0.001	0.002	0.002	0.002
	(0.0004)	(0.0004)	(0.001)	(0.001)
	$p = 0.005$	$p < 0.001$	$p < 0.001$	$p = 0.005$
Intercept	0.199	0.106	0.188	0.161
	(0.014)	(0.014)	(0.016)	(0.013)
	$p < 0.001$	$p < 0.001$	$p < 0.001$	$p < 0.001$
Observations	572621	417597	786620	141135
Log Likelihood	−980931	−693475	−1640532	−223934

Table S4: Mixed effects models predicting February-May 2020 changes in the number of submissions of research papers. The baseline is represented by the average of corresponding months in 2018 and 2019. Random intercepts included for countries.
