# Supplementary material for: Gender gap in journal submissions and peer review during the first wave of the COVID-19 pandemic. A study on 2329 Elsevier journals
Source: PLoS One. 2021 Oct 20;16(10):e0257919. doi: 10.1371/journal.pone.0257919 (PMC8528305; doi:10.1371/journal.pone.0257919)
Supplement: S5 Table — The baseline is represented by the average of corresponding months in 2018 and 2019. Random intercepts included for countries. (PDF) [file pone.0257919.s006.pdf]

	Health & Medicine	Life Sciences	Physical Sciences & Engineering	Social Sciences & Economics
Women	−0.105	−0.063	−0.075	−0.065
	(0.007)	(0.009)	(0.009)	(0.012)
	$p < 0.001$	$p < 0.001$	$p < 0.001$	$p < 0.001$
Age	−0.003	−0.001	−0.002	−0.003
	(0.0002)	(0.0003)	(0.0003)	(0.0004)
	$p < 0.001$	$p = 0.075$	$p < 0.001$	$p < 0.001$
Women×Age	0.001	0.001	0.002	0.002
	(0.0004)	(0.001)	(0.001)	(0.001)
	$p = 0.017$	$p = 0.005$	$p = 0.002$	$p = 0.036$
Intercept	0.229	0.136	0.182	0.163
	(0.014)	(0.015)	(0.016)	(0.014)
	$p < 0.001$	$p < 0.001$	$p < 0.001$	$p < 0.001$
Observations	345053	232588	659082	109992
Log Likelihood	−548169	−373027	−1339275	−164240

Table S5: Mixed effects models predicting February-May 2020 changes in the number of submissions of manuscripts submitted to Q1 journals. The baseline is represented by the average of corresponding months in 2018 and 2019. Random intercepts included for countries.
