# Supplementary material for: Gender gap in journal submissions and peer review during the first wave of the COVID-19 pandemic. A study on 2329 Elsevier journals
Source: PLoS One. 2021 Oct 20;16(10):e0257919. doi: 10.1371/journal.pone.0257919 (PMC8528305; doi:10.1371/journal.pone.0257919)
Supplement: S6 Table — The baseline is represented by the average of corresponding months in 2018 and 2019. Random intercepts included for countries. (PDF) [file pone.0257919.s007.pdf]

	Health & Medicine	Life Sciences	Physical Sciences & Engineering	Social Sciences & Economics
Women	−0.117	−0.038	−0.035	−0.043
	(0.010)	(0.010)	(0.010)	(0.016)
	$p < 0.001$	$p < 0.001$	$p = 0.001$	$p = 0.007$
Age	−0.002	−0.002	−0.006	−0.003
	(0.0004)	(0.0004)	(0.0004)	(0.001)
	$p < 0.001$	$p < 0.001$	$p < 0.001$	$p < 0.001$
Women×Age	−0.0004	−0.0005	−0.001	0.0003
	(0.001)	(0.001)	(0.001)	(0.001)
	$p = 0.604$	$p = 0.535$	$p = 0.247$	$p = 0.782$
Intercept	0.273	0.112	0.140	0.148
	(0.017)	(0.014)	(0.013)	(0.015)
	$p < 0.001$	$p < 0.001$	$p < 0.001$	$p < 0.001$
Observations	185966	114009	291372	54379
Log Likelihood	−301882	−169740	−532651	−80448

Table S6: Mixed effects models predicting February-May 2020 changes in the number of submissions by first authors. The baseline is represented by the average of corresponding months in 2018 and 2019. Random intercepts included for countries.
