# Supplementary material for: Gender gap in journal submissions and peer review during the first wave of the COVID-19 pandemic. A study on 2329 Elsevier journals
Source: PLoS One. 2021 Oct 20;16(10):e0257919. doi: 10.1371/journal.pone.0257919 (PMC8528305; doi:10.1371/journal.pone.0257919)
Supplement: S7 Table — The baseline is represented by the average of corresponding months in 2018 and 2019. Random intercepts included for countries. (PDF) [file pone.0257919.s008.pdf]

| | Health & Medicine | Life Sciences | Physical Sciences & Engineering | Social Sciences & Economics |
|---|---|---|---|---|
| Women | $-0.257$ | $-0.113$ | $-0.065$ | $-0.123$ |
| | (0.040) | (0.051) | (0.045) | (0.036) |
| | $p < 0.001$ | $p = 0.029$ | $p = 0.148$ | $p = 0.001$ |
| Age | $-0.006$ | $-0.005$ | $-0.006$ | $-0.002$ |
| | (0.001) | (0.001) | (0.001) | (0.001) |
| | $p < 0.001$ | $p < 0.001$ | $p < 0.001$ | $p = 0.085$ |
| Women×Age | 0.007 | 0.003 | $-0.001$ | 0.001 |
| | (0.002) | (0.003) | (0.003) | (0.003) |
| | $p = 0.003$ | $p = 0.315$ | $p = 0.717$ | $p = 0.792$ |
| Intercept | 0.463 | 0.160 | 0.171 | 0.118 |
| | (0.030) | (0.032) | (0.024) | (0.023) |
| | $p < 0.001$ | $p < 0.001$ | $p < 0.001$ | $p < 0.001$ |
| Observations | 16861 | 8983 | 33003 | 14015 |
| Log Likelihood | $-29182$ | $-15152$ | $-67330$ | $-22001$ |

Table S7: Mixed effects models predicting February-May 2020 changes in the number of submissions by solo authors. The baseline is represented by the average of corresponding months in 2018 and 2019. Random intercepts included for countries.
