# Supplementary material for: Gender gap in journal submissions and peer review during the first wave of the COVID-19 pandemic. A study on 2329 Elsevier journals
Source: PLoS One. 2021 Oct 20;16(10):e0257919. doi: 10.1371/journal.pone.0257919 (PMC8528305; doi:10.1371/journal.pone.0257919)
Supplement: S8 Table — The baseline is represented by the average of the corresponding months in 2019. Models included time in residential areas from Google’s COVID-19 Community Mobility Report (see https://www.google.com/covid19/mobility/; accessed on 30 June 2020). Random intercepts included for countries. (PDF) [file pone.0257919.s009.pdf]

	Health & Medicine	Life Sciences	Physical Sciences & Engineering	Social Sciences & Economics
Women	−0.007	0.017	0.002	−0.064
	(0.014)	(0.016)	(0.011)	(0.025)
	p = 0.613	p = 0.280	p = 0.860	p = 0.012
Age	0.002	0.002	0.001	0.001
	(0.0002)	(0.0002)	(0.0001)	(0.0003)
	p < 0.001	p < 0.001	p < 0.001	p = 0.004
Residential	0.002	0.001	0.001	−0.001
	(0.001)	(0.001)	(0.0005)	(0.001)
	p = 0.005	p = 0.264	p = 0.065	p = 0.197
Women×Age	0.0003	0.0001	−0.00004	0.001
	(0.0003)	(0.0003)	(0.0003)	(0.001)
	p = 0.336	p = 0.765	p = 0.895	p = 0.351
Women×Residential	−0.001	−0.001	−0.001	0.003
	(0.001)	(0.001)	(0.001)	(0.002)
	p = 0.569	p = 0.223	p = 0.438	p = 0.057
Intercept	−0.093	−0.096	−0.063	−0.025
	(0.010)	(0.012)	(0.007)	(0.014)
	p < 0.001	p < 0.001	p < 0.001	p = 0.065
Observations	86802	71101	163760	27005
Log Likelihood	−53020	−44604	−97616	−18430

Table S8: Mixed effects models predicting February-May 2020 changes in the proportion of accepted review invitations per area of research. The baseline is represented by the average of the corresponding months in 2019. Models included time in residential areas from Google's COVID-19 Community Mobility Report (see: `https://www.google.com/covid19/mobility/`; accessed on 30 June 2020). Random intercepts included for countries.
