# Supplementary material for: Gender gap in journal submissions and peer review during the first wave of the COVID-19 pandemic. A study on 2329 Elsevier journals
Source: PLoS One. 2021 Oct 20;16(10):e0257919. doi: 10.1371/journal.pone.0257919 (PMC8528305; doi:10.1371/journal.pone.0257919)
Supplement: S9 Table — The baseline is represented by the average of corresponding months in 2018 and 2019. Random intercepts included for countries. Gender data based on the stricter version of the gender guessing algorithm. (PDF) [file pone.0257919.s010.pdf]

	Health & Medicine	Life Sciences	Physical Sciences & Engineering	Social Sciences & Economics
Women	−0.181	−0.083	−0.099	−0.076
	(0.008)	(0.008)	(0.010)	(0.013)
	$p < 0.001$	$p < 0.001$	$p < 0.001$	$p < 0.001$
Age	−0.001	−0.002	−0.003	−0.003
	(0.0003)	(0.0002)	(0.0003)	(0.0004)
	$p < 0.001$	$p < 0.001$	$p < 0.001$	$p < 0.001$
Women×Age	0.001	0.002	0.002	0.002
	(0.0004)	(0.0004)	(0.001)	(0.001)
	$p = 0.005$	$p < 0.001$	$p < 0.001$	$p = 0.004$
Intercept	0.349	0.142	0.211	0.179
	(0.020)	(0.015)	(0.019)	(0.015)
	$p < 0.001$	$p < 0.001$	$p < 0.001$	$p < 0.001$
Observations	574167	368570	609207	117618
Log Likelihood	−1111722	−626152	−1285758	−187267

Table S9: Mixed effects models predicting February-May 2020 changes in the number of submissions per area of research area. The baseline is represented by the average of corresponding months in 2018 and 2019. Random intercepts included for countries. Gender data based on the stricter version of the gender guessing algorithm.
