# Supplementary material for: Gender gap in journal submissions and peer review during the first wave of the COVID-19 pandemic. A study on 2329 Elsevier journals
Source: PLoS One. 2021 Oct 20;16(10):e0257919. doi: 10.1371/journal.pone.0257919 (PMC8528305; doi:10.1371/journal.pone.0257919)
Supplement: S10 Table — The baseline is represented by the average of the corresponding months in 2019. Random intercepts included for countries. Gender data based on the stricter version of the gender guessing algorithm. (PDF) [file pone.0257919.s011.pdf]

	Health & Medicine	Life Sciences	Physical Sciences & Engineering	Social Sciences & Economics
Women	−0.015	−0.004	−0.005	−0.016
	(0.007)	(0.009)	(0.006)	(0.014)
	$p = 0.040$	$p = 0.644$	$p = 0.430$	$p = 0.232$
Age	0.002	0.002	0.001	0.001
	(0.0002)	(0.0002)	(0.0001)	(0.0004)
	$p < 0.001$	$p < 0.001$	$p < 0.001$	$p = 0.049$
Women×Age	0.0002	0.0002	−0.0001	0.0004
	(0.0003)	(0.0004)	(0.0003)	(0.001)
	$p = 0.574$	$p = 0.554$	$p = 0.715$	$p = 0.624$
Intercept	−0.064	−0.083	−0.046	−0.031
	(0.005)	(0.006)	(0.003)	(0.008)
	$p < 0.001$	$p < 0.001$	$p < 0.001$	$p < 0.001$
Observations	79235	65788	155981	23398
Log Likelihood	−48434	−41126	−93111	−15949

Table S10: Mixed effects models predicting February-May 2020 changes in the proportion of accepted review invitations per area of research. The baseline is represented by the average of the corresponding months in 2019. Random intercepts included for countries. Gender data based on the stricter version of the gender guessing algorithm.
