# Supplementary material for: Gender gap in journal submissions and peer review during the first wave of the COVID-19 pandemic. A study on 2329 Elsevier journals
Source: PLoS One. 2021 Oct 20;16(10):e0257919. doi: 10.1371/journal.pone.0257919 (PMC8528305; doi:10.1371/journal.pone.0257919)
Supplement: Attachment — Submitted filename: PONE2021RevisionResponseLetter.docx [file pone.0257919.s012.docx]

Reviewers' comments:

Reviewer's Responses to Questions

**Comments to the Author**

1. Is the manuscript technically sound, and do the data support the conclusions?

The manuscript must describe a technically sound piece of scientific research with data that supports the conclusions. Experiments must have been conducted rigorously, with appropriate controls, replication, and sample sizes. The conclusions must be drawn appropriately based on the data presented.

Reviewer #1: Yes

Reviewer #2: Yes

2. Has the statistical analysis been performed appropriately and rigorously?

Reviewer #1: Yes

Reviewer #2: Yes

3. Have the authors made all data underlying the findings in their manuscript fully available?

The [PLOS Data policy](C:\\C:\\Users\\MehmaniB\\Downloads\\1) requires authors to make all data underlying the findings described in their manuscript fully available without restriction, with rare exception (please refer to the Data Availability Statement in the manuscript PDF file). The data should be provided as part of the manuscript or its supporting information, or deposited to a public repository. For example, in addition to summary statistics, the data points behind means, medians and variance measures should be available. If there are restrictions on publicly sharing data—e.g. participant privacy or use of data from a third party—those must be specified.

Reviewer #1: Yes

Reviewer #2: Yes

4. Is the manuscript presented in an intelligible fashion and written in standard English?

PLOS ONE does not copyedit accepted manuscripts, so the language in submitted articles must be clear, correct, and unambiguous. Any typographical or grammatical errors should be corrected at revision, so please note any specific errors here.

Reviewer #1: Yes

Reviewer #2: Yes

5. Review Comments to the Author

Please use the space provided to explain your answers to the questions above. You may also include additional comments for the author, including concerns about dual publication, research ethics, or publication ethics. (Please upload your review as an attachment if it exceeds 20,000 characters)

Reviewer #1: This manuscript investigates the gap between female and male scientists in terms of their manuscript submissions and review activities under the current COVID-19 pandemic, using data from 2329 Elsevier journals. The study is innovative because it used rarely seen large-scale data for both manuscript submissions and peer reviews.

Additional comments:
(1) The manuscript does not have a literature review section. Some related work was mentioned in the introduction section of the manuscript, but providing additional literature survey will allow the readers to understand the topic much better.

*We have improved the background section by adding further (and more recent) references and elaborating more on the context and purposes of the study.*

(2) The overall data used in the study was from Feb-May 2018 to 2020. For comparisons between women and men’s submission, Feb-May 2019 and 2020 was used. For most countries, except China, social distancing measures and school closures did not happen until the middle of March (e.g. U.S.). Additionally, considering that for most of the manuscripts submitted in or before May, much of the work would have happened way before the world “shut down” due to the pandemic in most of the countries. Therefore, the time span used in the analysis is a little problematic. It will be useful to know what was the annual increase rate for submissions and reviews in Elsevier journals before the pandemic to rule out the possibility that the 30% increase in submissions between Feb-May 2019 and 2020 are in fact not due to normal annual increase.

*Good point. Figure 1 shows a smaller increase (11.7%) in submissions between 2018 and 2019, with a clear discontinuity in 2020 (+ 30.1 %). This corroborates the idea of looking at 2020 compared to the previous years as an exceptional period. We have added a reference to this discontinuity in the text. As regards the time window, by covering the whole period of March-May, we captured the effect of restrictions in EU and US, whereas with Feb 2020, we capture China and other Asian countries. Obviously, weekly instabilities could be present in the temporal pattern in specific places, but considering the whole period was instrumental to capture the big picture while considering country-effects. Note that from June on, anti-contagion measures were removed in many countries. So, extending the period to further months in the year would create other problems.*

(3) For results in Table S4, the text in the manuscript says the dependent variable is the change in submissions for individual academics, while the supplemental materials indicate that the dependent variable is the number of submissions. The inconsistency needs to be adjusted.

*The dependent variable in all models (both main text and SI) is the changes in the number of submissions. This is clearly indicated in the Table captions. Tab. S1 and S2 present descriptive statistics on the total number of submissions. This is also clearly indicated in the tables.*

(4) On page 6: “in addition, we ….with more senior cohorts of women less penalized than younger scholars.” I am having hard time concluding this from the results in Table S4. I think it will be better if authors can explain a little bit more here.

*We have improved the clarity of this part.*

(5) What is the percentage of “not title”, “doctor”, and “professor” category in the database? It will be useful to know in order to access the robustness of the analysis

*We have reported detail by adding the percentages in the text.*

(6) In all the analysis, how were those researchers entered “publishing” during the pandemic handled, considering they did not have publication histories prior to this? For the analysis on COVID-19 related submissions, this will be a bigger issue since there are a lot of people who did not publish on coronavirus related topics at all before they did so for COVID-19, which is particularly true of those non-biomedical COVID-19 publications.

*Datasets (both global one and the COVID-19 ones) include all scientists who submitted at least on paper in the feb-may period of any of the three years. This could be an issue for covid-related papers indeed, since this was a minor topic before 2020. However, the keywords used by Elsevier to classify the COVID related papers, which are fully reported in the Methods section, include keywords that help to classify all coronaviruses, SARS (severe acute respiratory syndromes) and alikes before COVID 19 that can reflect the involvement of a scholar in this type of research before the explosion of the COVID pandemic. On the other hand, the alternative solution of including only authors who submitted in 2020 would have biased the results in the opposite direction, in the sense that it would have artificially inflated the 2020 increase in the number of submission (point 2 above). Note that our decision as to map the* ***relative*** *differences between men and women. Therefore, any increase regarding both groups (eventually driven by newcomers – those who submitted a manuscript in 2020 who did not in 2019 and 2018) is considered by the model intercept, which is not subject to any interpretation in the text. The relative difference between men and women is captured by the gender coefficient, which is the one we actually discuss in the text. These controls for the overall increase allowed us to directly test the hypothesis of women penalization (relatively to men) in terms of manuscript submissions.*

(7) Based on the regression analysis results, the increase of submissions from female authors were smaller than that from male authors.

*The referee is absolutely right. We have clarified this point when presenting Figure 1.*

**Reviewer #2:**

I find the paper very interesting, and I think the penalisation of (especially young) women is indisputable in all areas, including academia. A supporting analysis definitely deserves attention.

Concerning the methodology, I must say that I am not an expert in linear mixed models, but I have some general comments.

The first critical point concerns the data used. In a first analysis, to estimate the gender effects of the pandemic on submissions, the authors calculate the submission difference index for each author (dependent variables) as the difference between new submissions February-May 2020 minus the average number of submissions for the same months and for the same author in the periods 2018 and 2019. I wonder how plausible it is for the same author to submit an article in the same months in the previous two years. I think the presence of unobservable variables is not taken into much consideration.

*Other studies trying to estimate pre-post COVID used gender differences in the proportion of authors submitting to journals because they did not have any possibility to control for individuals in different time periods. Therefore, they neglected the in-out flow of different authors. We discussed this alternative (i.e., measuring the differences in the proportion of men and women authors in a comparable time period) but concluded that given that we had the possibility to reconstruct individual trajectories (the same individual in a three-year time series), our measurements were more precise. This required to keep a comparable Feb-May time window. First, this allowed us to capture seasonality effects. Secondly, if we would have considered other months, we should have been required to make assumptions on differences between numbers of submissions per authors per months. This would have super-complicated our analysis adding further layers of assumptions. Obviously, in these types of analysis, the size and potential effect of unobservable variables are an important problem, but this is often unavoidable with analysis on large-scale data on human behaviour.*

Moreover, February-May 2020 seems to me to be a very limited period, but above all too "early", to see the effects on scientific production, especially in the West. Only in Italy, one of the first countries to be affected, have there been closures since March. In this regard, the authors control for different "geographical" measures of prevention by including random effects for countries.

*You are right. Indeed, we included random effects on countries to take into account a number of unobserved variables, along with the grouping of our observation within distinct cultural and institutional environments. Also note that the period under consideration covers well the restriction that were implemented during the first COVID wave at least in East Asia (China, Korea, ...), Europe, and North America.*

It is very interesting when they consider the scientific production on Covid-19 topics only, which surely has more concise working times, given the need of researchers to submit quickly. In that case, considering the time span February (maybe a bit early) - May seems to me more plausible.

*You are right. We plan to extend our observation scales in future research. But, to reflect a point discussed also by Referee 1, adding more months in the summer 2020 could create other problems (given the easining of the anti-contagion measures in certain countries). We are negotiating with Elsevier to extend the observation periods to June 2021 in order to capture a richer time windows and more importantly measure the reviewer and editorial decisions. More than extending the observation period to map manuscript submissions, we believe that a temporal extension is required to understand the effect of peer review and editorial decisions. In any case, you should imagine how much it’s difficult to go back to manuscript submission systems, extract further data (which are incredibly dirty), clean them, enrich them with gender, seniority and other variables. We believe this paper is the first milestone of a line of research that we hope to develop even more in the future.*

Furthermore, although it is clear that the focus of the paper is certainly not methodological, I think that a section (also in the supplementary materials) in which to motivate why linear mixed models have been considered and a minimum of formalisation is necessary. In addition, there is no test to verify the assumptions of the model (e.g. homogeneity, normality of error term, normality of random effect). Tests have probably been done, but this should at least be mentioned.

*Thank you for your suggestion. We have added a section on data analysis where we explicitly mention that standard tests were performed to check the model assumptions (homogeneity, normality of random effect, etc.) for all models, if not explicitly otherwise mentioned in the text.*
